# Passive adaptive grippers: a mini-review

**DOI:** 10.3389/frobt.2026.1747157

**Published:** 2026-05-07

**Authors:** Ming Chun Chan, Rob B. N. Scharff

**Affiliations:** 1 Division of Integrative Systems and Design, The Hong Kong University of Science and Technology, Kowloon, Hong Kong SAR, China; 2 Department of Mechanical and Aerospace Engineering, The Hong Kong University of Science and Technology, Kowloon, Hong Kong SAR, China

**Keywords:** adaptive grasping, embodied intelligence, passive grippers, soft grippers, soft robotics

## Abstract

Passive adaptive grippers leverage existing degrees of freedom (DOFs) of an external host system such as a robotic arm to complete a manipulation task. These grippers commonly rely on embodied intelligence to achieve this goal, leveraging interaction between the gripper and the environment to trigger prehension, retention, and release of an object. This mini-review establishes a framework for classification of state-of-the-art passive gripper designs across three phases of the gripping procedure: passive prehension (contact-loaded or preloaded), passive retention (externally or internally-sustained), and passive release (contact-based or contactless). Hereby, this work aims to accelerate future research on passive adaptive grippers and provide guidance for application-specific gripper design. Fully passive grippers that simultaneously combine reliable prehension, internally-sustained retention, and contactless release remain scarce. A fundamental trade-off exists between the gripper’s controllability and the host system’s flexibility; optimal gripper design must therefore be tailored to the specific task and operational constraints. Another key challenge is to minimize the force required to be exerted on the object to activate passive prehension. A promising direction towards addressing this challenge is the development of passive preloading mechanisms.

## 1 Introduction

Adaptive grasping enables grippers to grasp objects of unknown shape, size, or position. This is traditionally achieved through the integration of sensors that enable a closed-loop control scheme (Jacobsen et al., 1986; Kawasaki et al., 1999; Butterfass et al., 2001). In contrast, underactuated grippers can passively adapt to an object, thereby eliminating the need for sensors and real-time closed-loop control schemes (Tran et al., 2025; Lu et al., 2021; Hua et al., 2022; Kang et al., 2021; Tlegenov et al., 2014). A popular type of underactuated gripper is the soft robotic gripper, where soft materials are deployed to realize adaptive behavior (Gunderman et al., 2022; Li et al., 2024; Yue et al., 2025; AboZaid et al., 2024), often taking inspiration from nature (Nguyen et al., 2023). However, the above-mentioned grippers all require at least one dedicated actuator to grasp, hold, and release an object, which hinders their deployment in challenging environments where low energy consumption, robustness to dirt and water, and low costs are critical.

Passive grippers are designed with the intention to completely eliminate the need for dedicated actuators by leveraging the existing degrees of freedom (DOFs) of an external host system such as a robotic arm (Nate et al., 2023; Chan et al., 2025; Yue et al., 2022), uncrewed aerial vehicle (UAV) (Hsiao et al., 2022b; Hsiao et al., 2022a; Lee et al., 2024; Roderick et al., 2021; Yang et al., 2025) or uncrewed underwater vehicle (UUV) to complete a manipulation task. Passive grippers commonly rely on embodied intelligence to complete a task, leveraging environmental interactions to trigger prehension, retention and release of an object. This concept allows robot behavior to emerge from the coupling of control, body morphology, and environment, thereby enabling passive mechanisms to outsource control to physical properties such as compliance and friction for efficient and adaptive grasping without centralized computation (Pfeifer et al., 2007).

Despite a growing body of research into passive adaptive grippers, a structured classification framework is still missing, hindering thorough understanding of critical design decisions and their functional implications. This mini-review addresses this gap by introducing a classification framework and performing a comprehensive mapping of the state-of-the-art onto this framework. Hereby, this work aims to accelerate future research and provide guidance for application-specific gripper design. First, Section 2 presents a classification of the mechanisms that have been deployed for passive adaptive prehension, passive retention, and passive release. Next, Section 3 highlights promising directions for future research based on the presented classification and applications. Finally, Section 4 concludes this mini-review by synthesizing the key findings.

## 2 A classification of passive grippers

**FIGURE 1:**
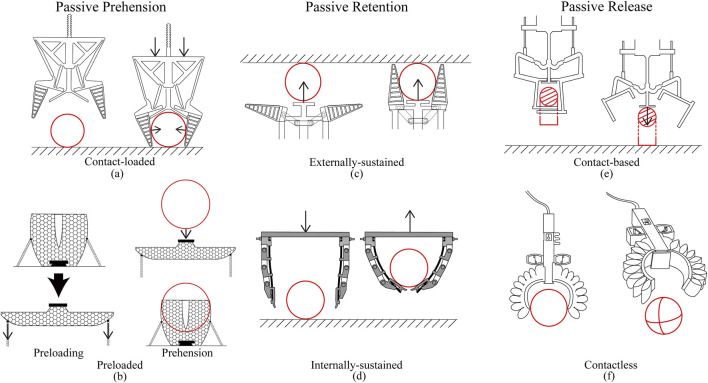
Framework for classification of passive adaptive grippers with examples: **(a)** contact-loaded prehension mechanism by Chan et al. (2025), **(b)** preloaded prehension mechanism by Hou et al. (2024), **(c)** externally-sustained retention mechanism by Lee et al. (2024), **(d)** internally sustained retention mechanism by Nate et al. (2023), **(e)** contact-based release mechanism by Hsiao et al. (2022a), and **(f)** contactless release mechanism by Zhai et al. (2023).

The functionality of a passive gripper is best understood by separately analyzing each phase of the gripping procedure: prehension, retention, and release. For passive prehension, a distinction is made between contact-loaded and preloaded grippers. Next, for passive retention, a distinction is made between externally and internally sustained retention. Finally, for passive release, a distinction is made between contact-based release and contactless release. The resulting classification framework is presented in Figure 1 and will be discussed in detail in this section.

Table 1 summarizes the key characteristics of the passive grippers found in the literature review. Here, passive prehension was used as the key inclusion criterion. Active grippers with passive retention or passive release (Crooks et al., 2017; Guo et al., 2020; Tran et al., 2025; Hsu et al., 2017; Gunderman et al., 2022; Yoon and Kim, 2022) were thus excluded from this review. As passive prehension and retention with contact-loaded suction cups is well-understood and extensively reported in literature, only preloaded suction cups (Yue et al., 2022) or suction cups with an additional passive retention mechanism (Hsiao et al., 2022b) were included in this review. Pneumatically actuated grippers with a contact-based trigger were included only if the system would still function if the pressure supply was replaced by a pressure vessel, making the system qualify as preloaded passive prehension. As a result, Zhai et al. (2023) was included in this review, whereas Yue et al. (2025) was excluded due to the system’s reliance on pumps. Finally, jamming grippers that passively conform to an object (Brown et al., 2010; Wang et al., 2025) but require application of a vacuum to complete prehension were also excluded from this review.

**TABLE 1:** Key characteristics of the passive adaptive grippers from the literature review.

Authors	Host platform	Gripper properties		Gripper phase		
		Grasp type	Material	Passive prehension	Passive retention	Passive release
Hsiao et al. (2022a)	UAV	Impactive	Hard	Yes, contact-loaded	Yes, internally-sustained	Yes, contact-based
Nate et al. (2023)	Robot arm	Impactive	Hard	Yes, contact-loaded	Yes, internally-sustained	Yes, contact-based
Lee et al. (2024)	UAV	Impactive	Soft	Yes, contact-loaded	Yes, externally-sustained	Yes, contactless
Chan et al. (2025)	Robot arm	Impactive	Soft	Yes, contact-loaded	Yes, internally-sustained	Yes, contactless
Liu et al. (2020)	Climbing robot	Ingressive	Hard	Yes, preloaded	Yes, internally-sustained	Yes, contactless
Zhai et al. (2023)	-	Impactive	Soft	Yes, preloaded	Yes, internally-sustained	Yes, contactless
Hsiao et al. (2022b)	UAV	Astrictive	Soft	Yes, contact-loaded	Yes, internally-sustained	No
Nagaoka et al. (2018)	Four-legged robot	Ingressive	Hard	Yes, contact-loaded	Yes, externally-sustained	No
Yue et al. (2022)	Robot arm	Astrictive	Soft	Yes, preloaded	Yes, internally-sustained	No
Petkovic et al. (2012)	-	Impactive	Soft	Yes, contact-loaded	Yes, externally-sustained	No
Hou et al. (2024)	-	Impactive	Soft	Yes, preloaded	Yes, internally-sustained	No
Roderick et al. (2021)	UAV	Impactive, ingressive	Hard	Yes, preloaded	Yes, internally-sustained	No
Wang et al. (2020)	-	Impactive	Soft	Yes, contact-loaded	Yes, internally-sustained	No
Yang et al. (2025)	UAV	Impactive	Soft	Yes, preloaded	Yes, internally-sustained	No
Seibel et al. (2020)	-	Contigutive	Soft	Yes, contact-loaded	Yes, internally-sustained	No
Hills et al. (2024)	-	Ingressive	Hard	Yes, contact-loaded	Yes, internally-sustained	No
Cai et al. (2025)	Robot arm	Impactive	Soft	Yes, preloaded	Yes, internally-sustained	No
Liu et al. (2023)	Robot arm	Impactive	Soft	Yes, preloaded	Yes, internally-sustained	No
Cai and Tang (2025)	-	Impactive	Soft	Yes, contact-loaded	Yes, internally-sustained	No
Lin et al. (2021)	Robot arm	Impactive	Soft	Yes, preloaded	Yes, internally-sustained	No

For each passive gripper in Table 1, we describe the host platform, the grasp type, the gripper materials to indicate the adaptiveness, and the grippers’ ability to passively prehend, retain, and release objects. Following the classification by Monkman et al. (2007), grasp types comprise impactive, ingressive, astrictive, and contigutive. The following paragraphs provide a detailed description of the mechanisms found to passively perform each phase of the gripping procedure.

### 2.1 Passive prehension

The passive grippers in Table 1 can be classified according to the way in which energy is loaded into the gripper: (1) contact-loaded mechanisms, where energy is transferred to the gripper during prehension via the application of a push force on the object along a displacement (see Figure 1a), and (2) preloaded mechanisms, where energy is stored prior to prehension (e.g., in springs or compressed air) and contact with the object triggers the release of this energy to activate prehension (see Figure 1b).

#### 2.1.1 Contact-loaded mechanisms

Contact-loaded prehension relies on the host system’s motion (e.g., a robotic arm or UAV) to generate a push force along a displacement that triggers the gripper’s grasping mechanism. Compliant mechanisms are commonly deployed in contact-loaded prehension, where the gripper’s structure deforms upon contact to envelop the object (Petkovic et al., 2012; Hills et al., 2024; Qi et al., 2022; Cai and Tang, 2025). Fin Ray fingers are a notable example. Lee et al. (2024) utilized Fin Ray fingers in a UAV-mounted gripper for perching on rod-like structures, where the UAV’s lifting force triggers deformation to passively grasp the object. With a UAV push force of around 7.84 N , the Fin Ray fingers provide around 3 N of grasping force. The compliant nature of Fin Ray fingers allows adaptive grasping of diverse shapes, but their effectiveness depends on object geometry. Similarly, Nate et al. (2023) presented a contact-loaded passive gripper that uses the mechanical work performed during ground contact to actuate its caging mechanism. This design is simple and energy-efficient but limited to caging grasps and unsuitable for soft objects. Hsiao et al. (2022a) also developed a mechanically intelligent UAV gripper that locks a compliant mechanism upon contact with objects, leveraging the UAV’s DOFs for passive prehension. The force required to close the gripper upon initial contact is approximately 0.42 N . However, this gripper is constrained to handling objects with holes or handles. Chan et al. (2025) developed a passive gripper using a compliant linkage to close a pair of Fin Ray fingers when the gripper is pushed onto an object (see Figure 1a). Contact-loaded passive grippers require the application of a push force on the object along a displacement. For example, the gripper by Chan et al. (2025) requires the application of a push force of 10 N to close the gripper, whereas the bistable mechanism by Hsiao et al. (2022a) requires the application of 4−8  N of push force to close the gripper. These forces hinder the grasping of delicate objects as well as highly compressible objects (e.g., a sponge) and loosely supported objects (e.g., an apple hanging on a tree).

#### 2.1.2 Preloaded mechanisms

Preloaded mechanisms store energy in the gripper’s structure (e.g., springs) and release it upon contact to trigger prehension, with the contact force serving as a trigger rather than the primary energy source. Hou et al. (2024) and Yang et al. (2025) developed passive preloaded tendon-net grippers inspired by fly-traps and spider webs, respectively. Hou et al. deployed a bistable origami structure with a preloaded tendon net that encloses the object upon contact and locks via snap-through, whereas Yang et al. attached four soft fingers that passively enclose the object upon contact (see Figure 1b). Similarly, Roderick et al. (2021) presented a bird-inspired gripper for perching on rod-like structures, where a preloaded spring in a tendon-driven mechanism is triggered by contact to close the gripper. This gripper is effective for perching but limited to rod-like objects and requires spring preloading prior to each grasp. Liu et al. (2020) presented a spine-based gripper for climbing robots, where spines powered by preloaded springs penetrate rough surfaces upon contact, enabling passive grasping for climbing. The gripper by Zhai et al. (2023) is preloaded using compressed air, with a contact switch triggering the release of the compressed air into a pair of soft pneumatic actuators to realize prehension. Yue et al. (2022) designed a preloaded suction cup that actively applies negative pressure to invert a toroidal-shaped snap-through membrane. Upon contact with the object, this stored elastic energy is released to realize prehension. Finally, Cai et al. (2025) developed a Kresling origami bistable gripper with electromagnetic actuation. The gripper is preloaded by a +17  V pulse and closes its snap-through mechanism when the trigger force exceeds 1.6  N . These preloaded systems offer rapid and adaptive responses which only require a small amount of energy to be applied to the object. For example, the energy required to trigger the gripper for Yang et al. (2025) and Hou et al. (2024) tendon nets is around 0.0438−0.4446  J and 0.0014  J respectively. The force required to trigger the contact switch of the pneumatic finger by Zhai et al. (2023) is approximately 0.14  N . This can effectively prevent excessive contact forces on delicate objects. However, preloaded systems typically require an actuator to reset the mechanism after completing the gripping procedure, making these systems not truly passive.

### 2.2 Passive retention

Once prehension is completed, the object can be passively retained in one of two ways: (1) externally-sustained retention, where the hold of the object is sustained through continued work performed by the host system or environment (see Figure 1c), and (2) internally-sustained retention, where the hold of the object can be maintained independently of the host system (see Figure 1d).

#### 2.2.1 Externally-sustained

Externally-sustained passive retention depends on sustained contact force from the object to maintain the gripper’s configuration. For example, Lee et al. (2024) developed a compliant Fin Ray gripper mounted on a UAV. Upon contact with an object, the push-rod triggers the compliant structure to enclose and hold the object. However, the UAV must continuously push upward to sustain the grasp, as retention relies on ongoing contact force between the push-rod and the object (see Figure 1c). The minimal force required to close the Fin Ray fingers of the gripper is 3.236 N . Nagaoka et al. (2018) developed a passive spine gripper for a four-limbed climbing robot, which holds itself using spines that hook onto rough surfaces. The gripper maintains its hold onto the rock for as long as the host robot pushes the gripper against the wall with a force of 3−7 N . These designs rely on ground reaction forces to exert a force on the object and thus struggle to lift an object from the ground for transport purposes.

#### 2.2.2 Internally-sustained

Internally-sustained passive retention is achieved through internal mechanical locking, eliminating dependency on continuous object contact. Once triggered, the gripper remains locked using stored energy or geometric constraints. Hsiao et al. (2022a) presented a compliant linkage gripper that self-locks after a push force is applied and remains locked until re-triggered. The self-locking mechanism can passively hold loads up to 3.7 kg . Hsiao et al. (2022b) also designed a passive suction cup that is triggered upon contact with a surface. After attachment, a bistable locking mechanism enables retention of heavy objects. Chan et al. (2025) designed a gripper with a push-rod and linear ratchet mechanism that locks upon contact and holds the object firmly without requiring additional energy or contact force. Nate et al. (2023) presented a passive eight-fingered gripper, where protrusions on the fingers interlock with a perforated plate to lock the fingers (see Figure 1d), thereby maintaining a grip without consuming energy. In the spine-based wall-climbing gripper introduced by Liu et al. (2020), spines are engaged through the release of a preloaded spring and remain locked until an automated mechanism resets the preload. Yang et al. (2025) developed a bistable gripper with a tension net that snaps into a stable closed state upon impact, using the internal energy barrier and tension net to retain objects even when contact pressure varies.

These grippers do not continuously require energy from the host system, thus offering greater energy efficiency. This makes them ideal for applications such as UAV perching, wall climbing, and aerial manipulation. Internally-sustained retention typically relies on mechanical interlocking or bistable structures that allow passive grippers to retain objects without requiring ground reaction forces, at the cost of adding mechanical complexity.

### 2.3 Passive release

Passive release is accomplished using the built-in actuation of the host system. Passive release mechanisms can be divided into two classes: (1) contact-based release mechanisms, where ground reaction forces are required to release the object (see Figure 1e), and (2) contactless release mechanisms, where the grippers are able to release the object in the air or without contact with the ground (see Figure 1f).

#### 2.3.1 Contact-based release

Contact-based passive release refers to a mechanism where the gripper releases an object by making contact with the ground or a wall to open and disengage the gripper. Hsiao et al. (2022a) developed a gripper that releases the object by pressing the base of the gripper against a surface to unlock the mechanism, allowing the object to be freed (see Figure 1e). Nate et al. (2023) designed a gripper capable of passive grasping, but release requires the gripper’s fingers to contact the ground again to unlock and disengage them.

As these grippers rely on contact force to trigger the unlocking of the retention mechanism, their operational range is limited. Additionally, effective obstacle avoidance is essential during operation to prevent unintended releases caused by accidental contact.

#### 2.3.2 Contactless release

Contactless passive release enables the gripper to detach an object without requiring contact with the ground or a wall. Lee et al. (2024) introduced a gripper mounted on a UAV that passively releases an object; by ceasing the upward thrust, the mechanism allows the Fin Ray fingers to open. Zhai et al. (2023) designed a contactless gripper that releases an object by tilting the gripper’s orientation. It relies on a gravity switch that cuts off the air supply to a pneumatic circuit, allowing pressurized air in the gripper fingers to release through the exhaust ports (see Figure 1f). Chan et al. (2025) presented a passive release mechanism that releases the object at a specific rotation of the gripper through gravity. This was achieved by an asymmetric wheel that pushes two spring-loaded pawls outwards as the gripper rotates, causing it to release the locking mechanism. These contactless passive release mechanisms eliminate the need for environmental contact, offering high flexibility in aerial or unstructured environments (e.g., mid-air delivery). However, these grippers also lack the ability to precisely place objects. Moreover, fragile objects may be damaged upon impact, making these grippers less suitable for fragile payloads.

## 3 Discussion

### 3.1 Design trade-off

As passive grippers inherently rely on the host system’s actuators to drive the gripping procedure, a trade-off exists between the gripper’s controllability and the host system’s flexibility. For a gripper to be considered passive, all of the host system’s DOFs should be deployable for maneuvering during at least one point in the gripping procedure. A key challenge in the design of passive grippers is therefore to determine which DOFs can be borrowed from the host system at what point in the gripping procedure to minimize the impact on the host system’s flexibility. For instance, Liu et al. (2020) developed a passive climbing robot driven by a single locomotion motor. While the strict coupling between the spiny grippers and the locomotion system preserves the flexibility of the host system, it offers no independent control over gripper prehension, retention, and release. In contrast, Chan et al. (2025) designed a fully passive gripper that requires interaction with the object to initiate prehension and leverages gravitational forces to passively release the object only when the gripper is maneuvered in a specific orientation, thus maintaining most of the host system’s DOFs for maneuvering the object in space during the retention phase.

### 3.2 Passive preloading

A limitation of contact-loaded passive grippers is that a push force needs to be exerted on the object to activate the prehension, which can damage delicate objects or even prevent the grasping of highly compressible and loosely supported objects. Preloaded mechanisms address this limitation. However, all preloaded mechanisms in this literature review required an actuator to apply the preload, making them not truly passive. Passive preloading mechanisms could be a promising direction to realize truly passive grippers capable of handling delicate objects. The host motion system could be leveraged as an energy source for the preloading of the grasping mechanism (e.g., compressing springs). For example, Bianchi et al. (2024) demonstrated that mechanical energy can be stored by performing circular run-ups with a soft robotic arm. Such principles could potentially be deployed to collect energy for preloading the prehension mechanism.

## 4 Conclusion

This mini-review introduced a framework for classifying passive adaptive grippers and performed a comprehensive mapping of the state-of-the-art onto this framework. Fully passive grippers that combine passive prehension, retention, and release remain scarce. A fundamental trade-off exists between the gripper’s controllability and the host system’s flexibility; optimal gripper design must therefore be tailored to the specific task and operational constraints. Whereas grippers with contact-loaded prehension mechanisms suffer from the requirement for relatively large forces to be exerted on the object, preloaded prehension mechanisms typically still require an actuator to reset the mechanism after completing the gripping procedure. A promising direction for future research is the use of the host system for preloading the gripper to realize truly passive grippers capable of handling delicate objects.
